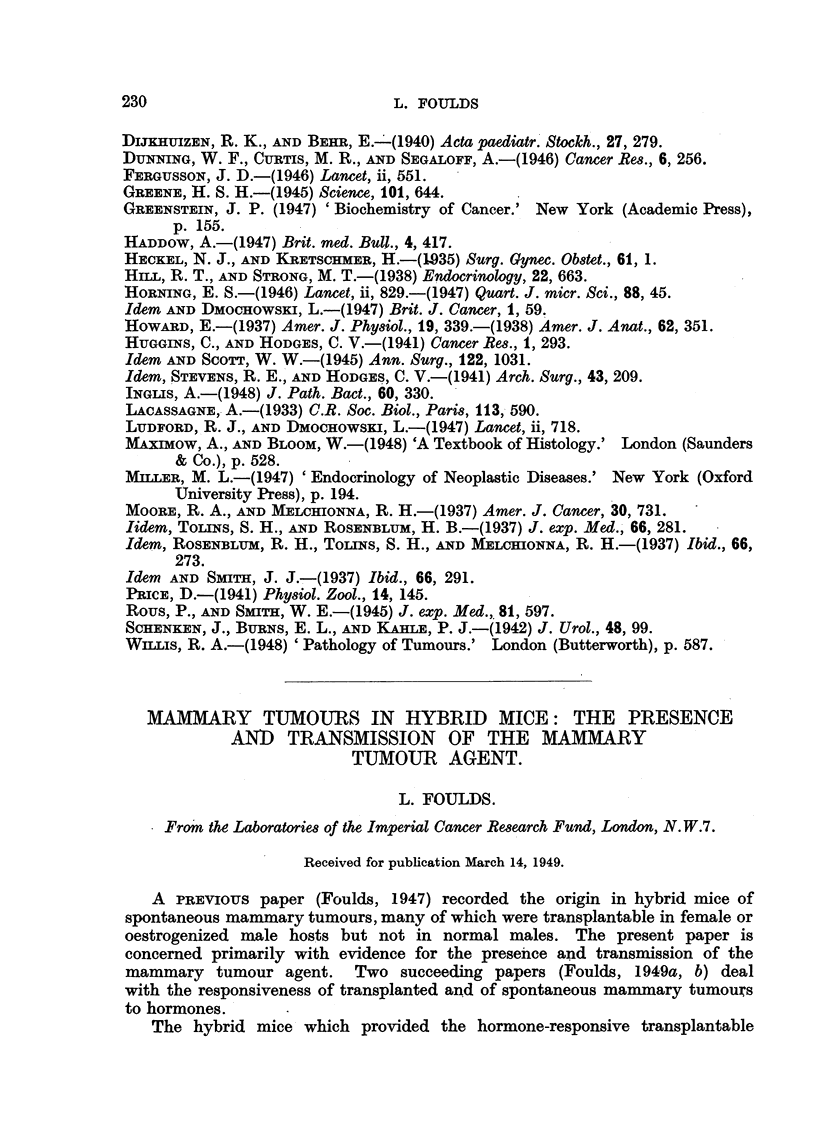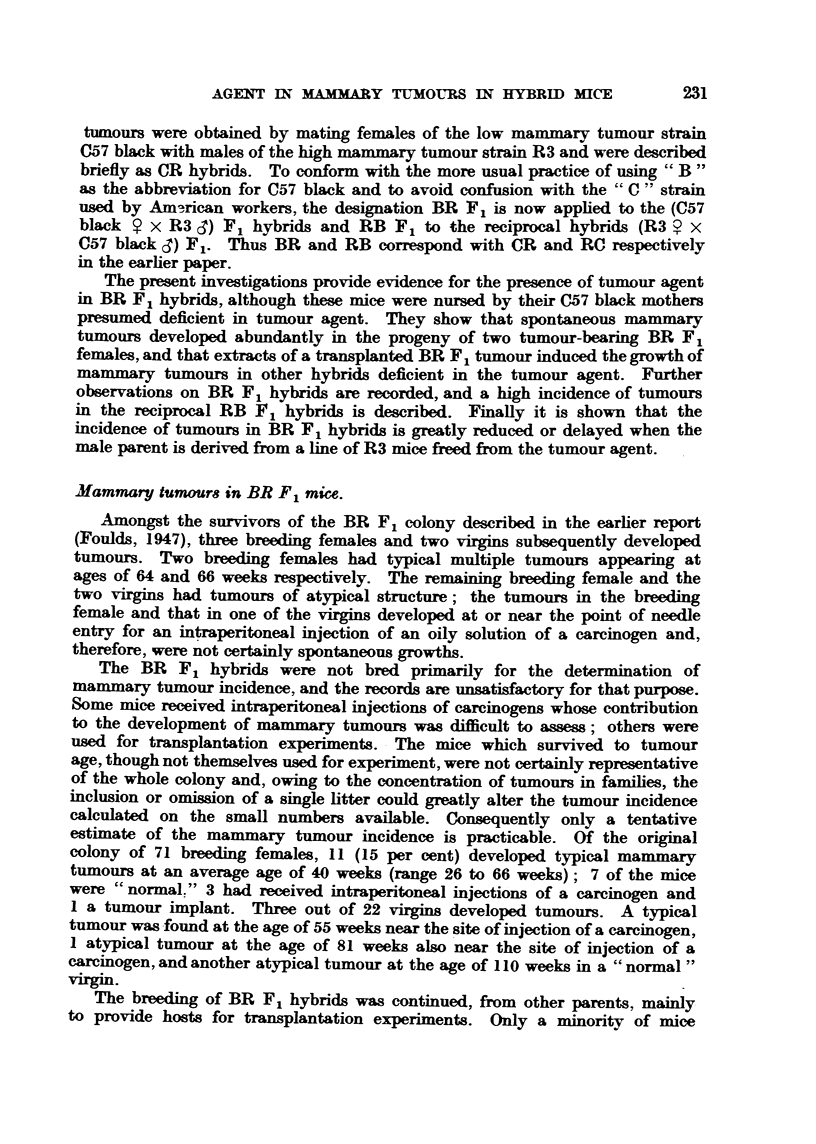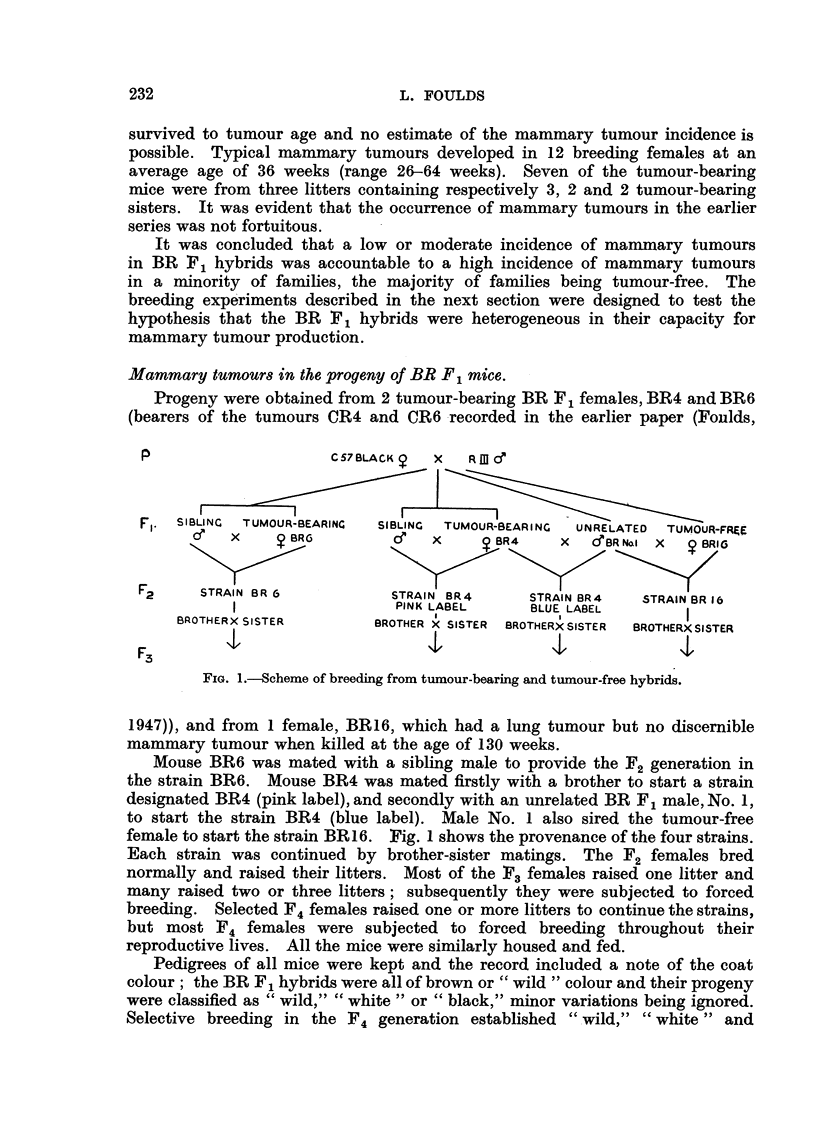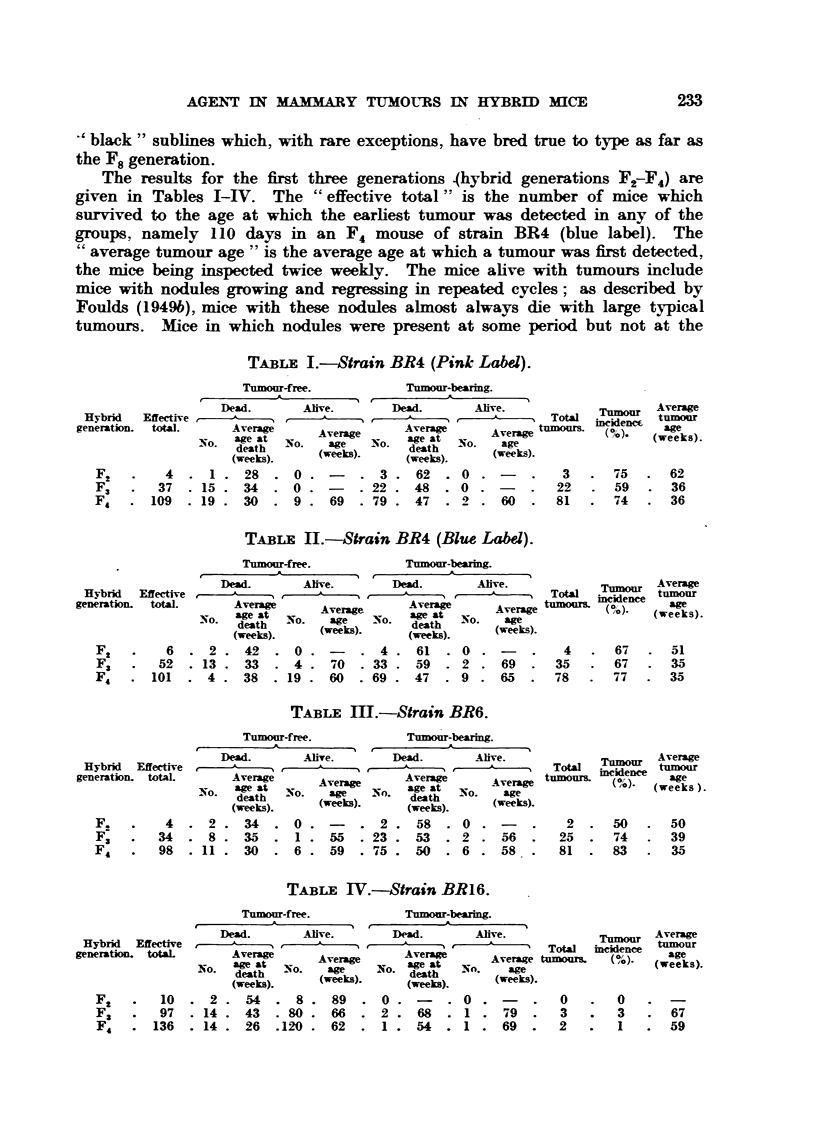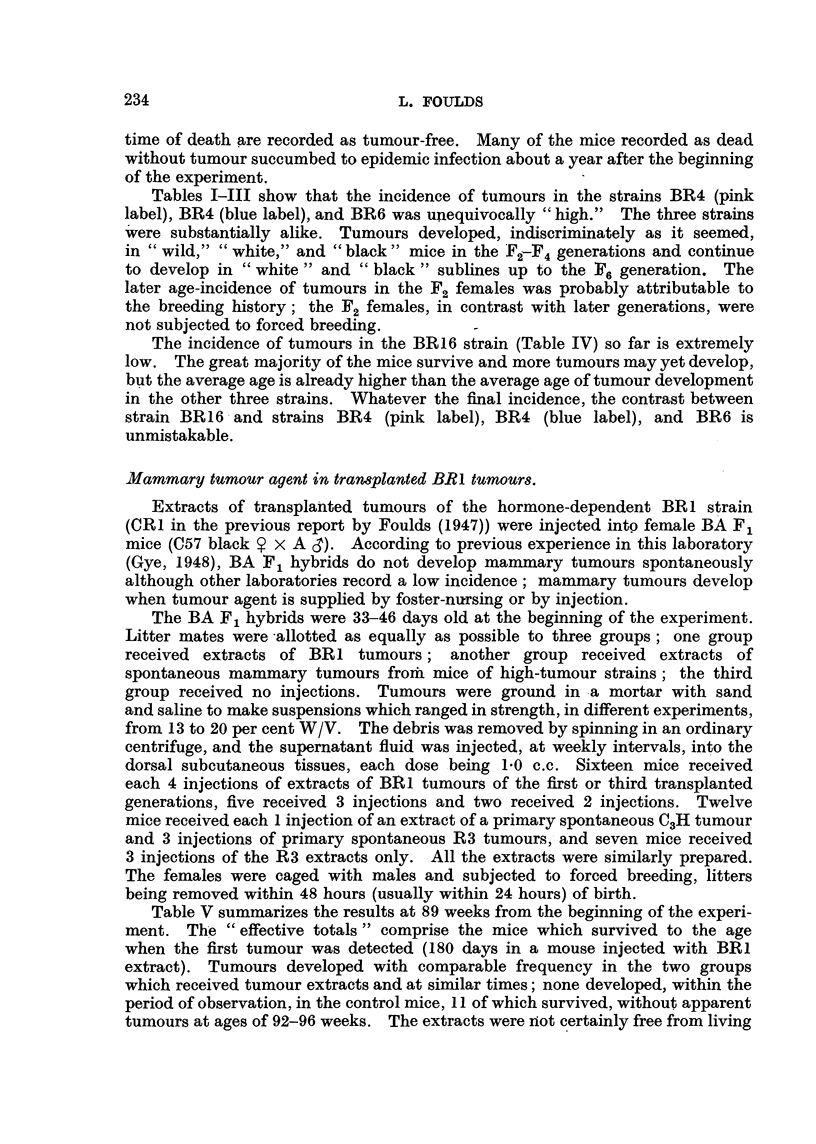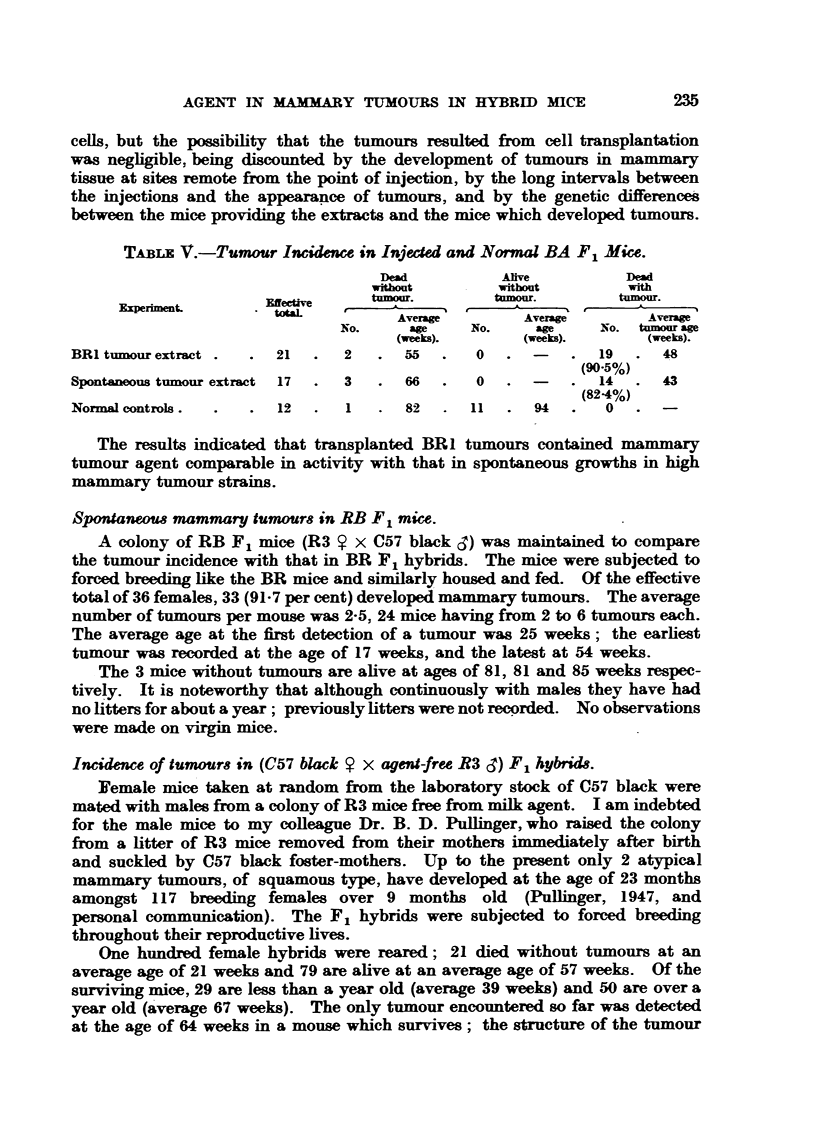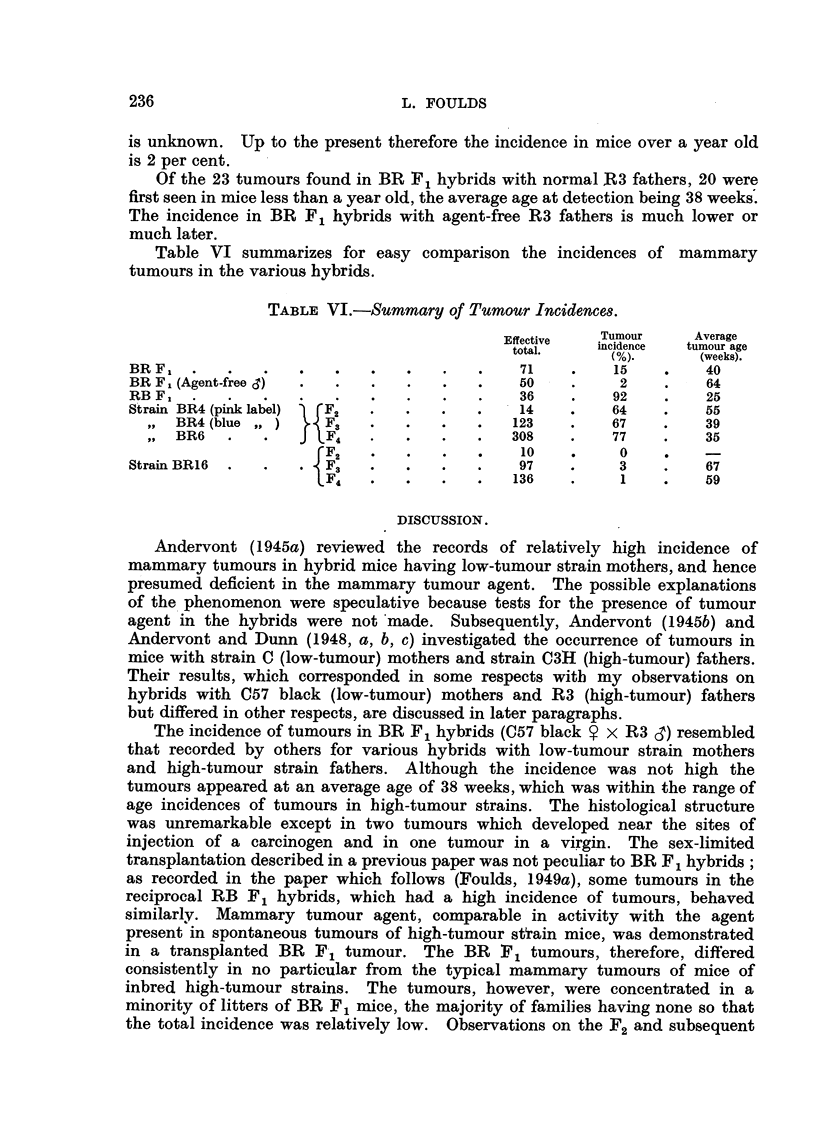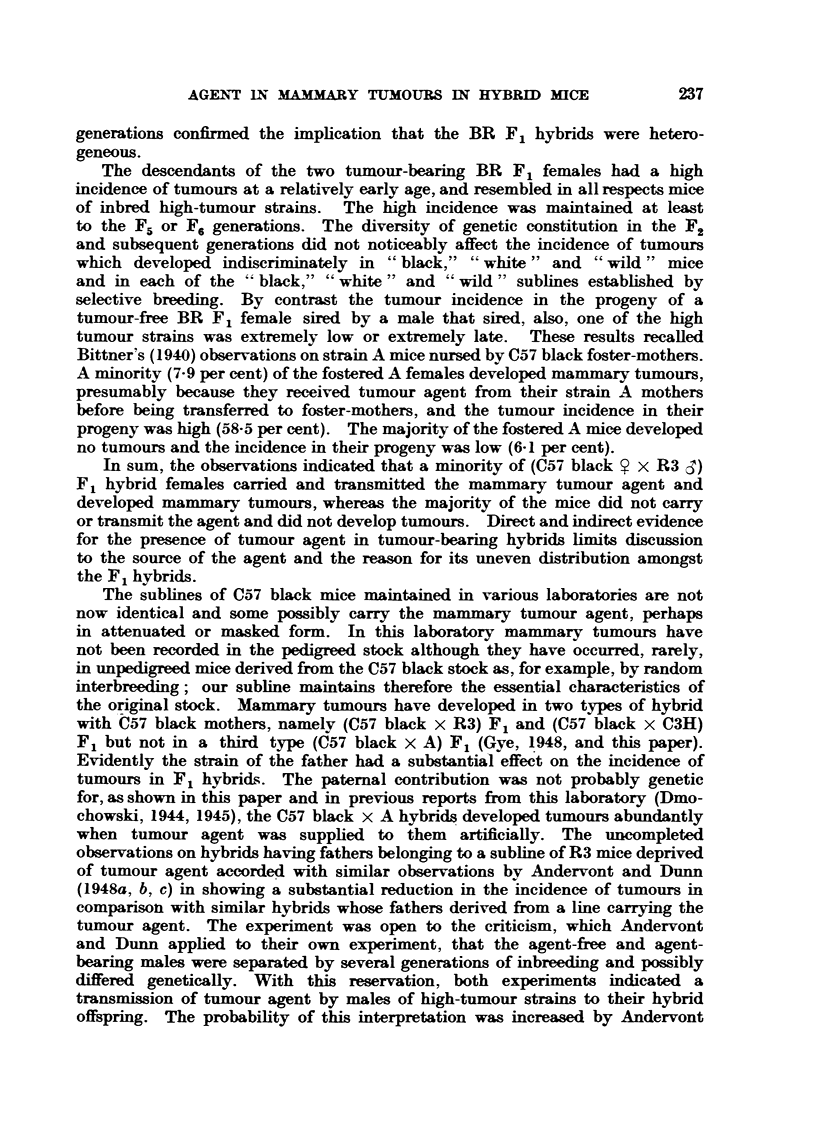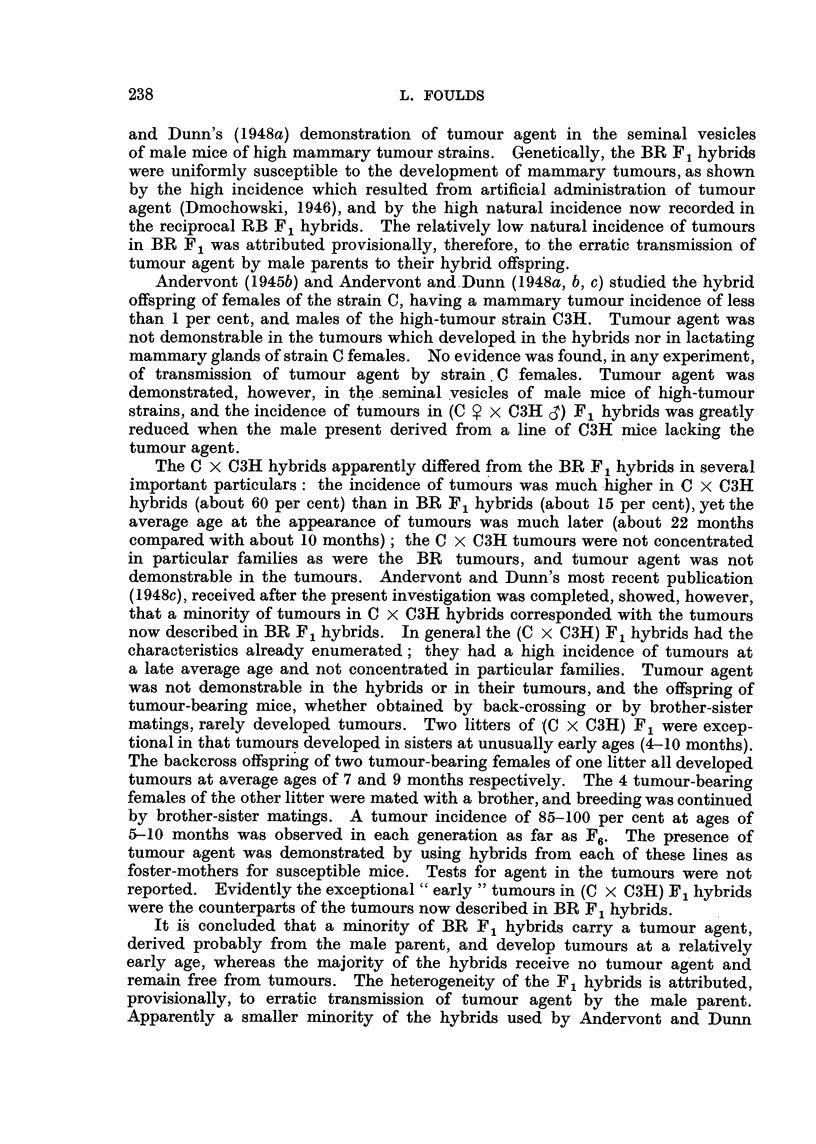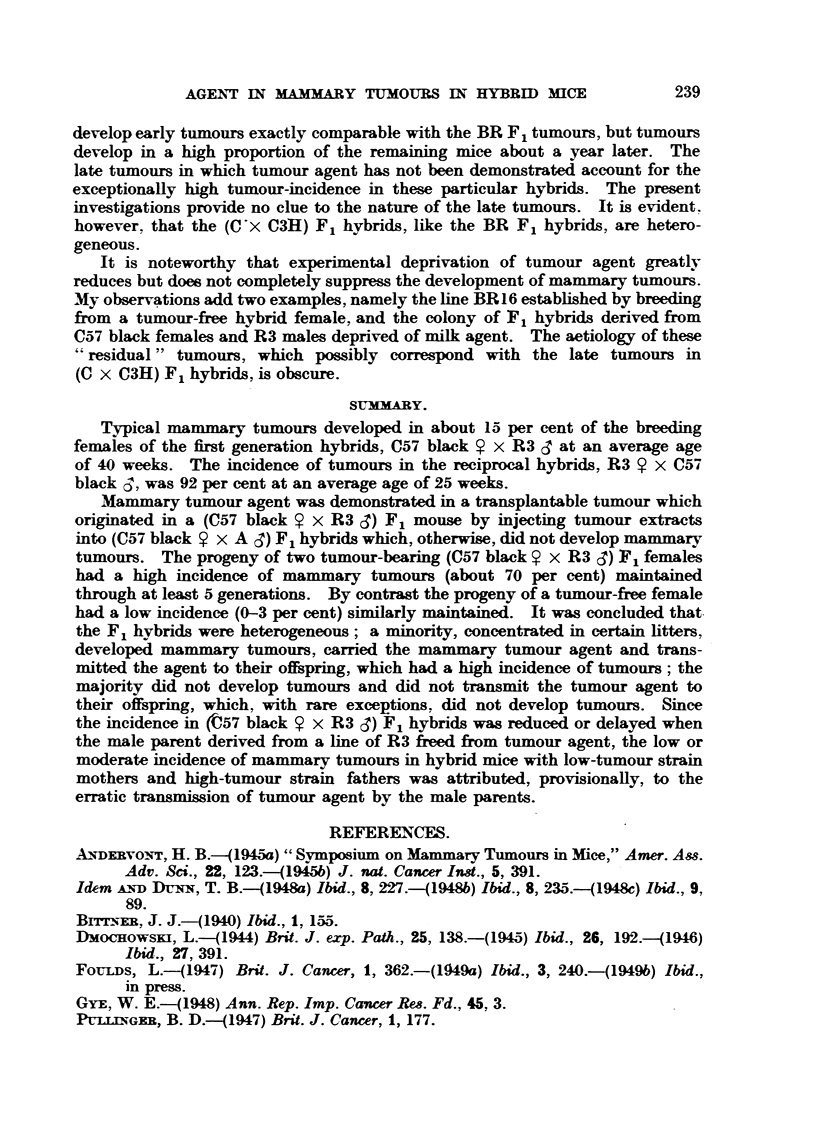# Mammary Tumours in Hybrid Mice: the Presence and Transmission of the Mammary Tumour Agent

**DOI:** 10.1038/bjc.1949.23

**Published:** 1949-06

**Authors:** L. Foulds


					
MAMMARY TUMOURS IN HYBRID MICE: THE PRESENCE

AND TRANSMISSION OF THE MAMMARY

TUMOUR AGENT.

L. FOULDS.

From W Laboratorie-8of the, Imperial Cancer Rmearch Fund, London, N. W-7.

Received for publication March 14, 1949.

A PREVIOUS paper (Foulds, 1947) recorded the origin in hybrid mice of
spontaneous mammary tumours, many of which were transplantable in female or
oestrogenized male hosts but not in normal males. The present paper is
concerned primarily with evidence for the prese-nee and transmission of the
mammary tumour agent. Two succeeding papers (Foulds, 1949a, b) deal
with the responsiveness of transp ante an o spontaneous mammary tumours
to hormones

The hybrid mice, which provided the hormone-responsive transplantable

O-Ql

APO

AGENT IN MAMMARY TU'MOURS IN HYBRID 3RCE

tumours were obtained by mating females of the low mamm ry tumour strain
C57 black with males of the   mammary tumour strain R3 and were described
briefly as CR hybrids. To conform with the more usual practice of using " B 93
a-s the abbreviation for C57 black and to avoid confusion with the "' C" strain
u-sed by Am-'rican workers, the designation BR F, is now apphed to the (C57
black Y x R3 S) F, hybrids and RB F, to the reciprocal hybrids (R3      x
C57 black cT) Fl. Thus BR and RB corrc-spond with CR and RC respectively
in the earher paper.

The prewnt investigations provide evidence for the presence of tumour agent
in BR F , hybrids, although these mice were nursed by their C5 7 black mothers
presumed deficient in tumour agent. They show that spontaneous mam iary
tumours developed abundantly in the progeny of two tuinour-bearing BR F,
females, and that extracts of a transplanted BR F 1 tumour induced the growth of
mammary tumours in other hybrids deficient in the tumour agent. Further
observations on BR F, hybcicls we xecorded, and a high incidence of tumours
in the reciprocal RB F, hybrids is described. Finally it is shown that the
incidence of tumours in BR F, hybrids is, greatly reduced or delayed when the
male parent is derived from a line of R3 mice fi-eed from the tumour agent.

Mammary tunwurs in BR F., mice.

Amongst the survivors of the BR F, colony described in the earher report
(Foulds, 1947), thxee breeding females and two virgins subsequently developed
tumours. Two breeding females had typical multiple tumours apWAring at
ages of 64 and 66 weeks revmtively. The re      i i  breeding female and the
two virgins had tumours of atypical structure; the tumours in the breeding
female and that in one of the virgins developed at or near the point of needle
entry for an intraperitoneal injection of an oily -solution of a carcinogen and,
therefore, were not certainly spontaneous growths.

The BR F,, hybrids were not bred primarily for the determination of
mammary tumour incidence, and the records are unsatisfactory for that purpow.
Some mice received intraperitoneal injec-tions of carcinogens whose contribution
to the development of mammary tumours wa-s difficult to aswss ; others were
used for transplantation experiments. - The mice which survived to tumour
age, though not themselves used for experiment, were not certainly representative
of the whole colony and, owing to the concentration of tumours in famihes, the
inclusion or omission of a single litter could greatly alter the tumour incidence
calculated on the small nurabers available. Conwquently only a tentative
estimate of the mammary tumour incidence is practicable. Of the original
colony of 71 breeding females, I I (15 per cent) developed typical mammary
tumours at an average age of 40 weeks (range 26 to 66 weeks); 7 of the mice
were   normal. 9' 3 had received intraperitoneal injecti  of a carcinogen and
I a tumour implant. Three out of 22 virgins developed tumours. A      ical
tumour was -found at the age of 55 weeks near the site of injection of a carcinogen,
I atypical tumour at the age of 81 weeks also near the site of injection of a
carcinogen, and another atypical tumour at the age of II 0 weeks in a "normal
virgin.

The breeding of BR F, hybrids was continued, from other parents, mainly
to provide hosu for transplantation experiments. Only a mi iority of mice

232

L. FOULDS

survived to tumour age and no estimate of the mammary tumour incidence is
possible. Typical mammary tumours developed in 12 breeding females at an
average age of 36 weeks (range 26--64 weeks). Seven of the tumour-bearing
mice were from three litters containing respectively 3, 2 and 2 tumour-bearing
sisters. It was evident that the occurrence of mammary tumours in the earlier
series was not fortuitous.

it was concluded that a low or moderate incidence of manmary tumours
in BR IF, hybrids was accountable to a high incidence of mammary tumours
in a niinority of farnilies, the majority of families being tumour-free. The
breeding experiments described in the next section were designed to test the
hypothesis that the BR F, hybrids were heterogeneous in their capacity for
mammary tumour production.

Mammary tumours in the progeny of BR F, mice.

Progeny were obtained from 2 tumour-bearing BR P 1 females, BR4 and BR6
(bearers of the tumours CR4 and CR6 -recorded in the earlier paper (Foulds,

CS78LACK     x   Rme

Fl. SIBLINC  TUMOUR-BEARINC,  SIBLING  TUMOUR-SEARING  UNRELATED  TUMOUR-FREF-

e    x      BRO         e    x       BR4     X   Cr BR Nal X  BRIG

F       STRAIN BR 6

2                             STRAIN BR4       STRAIN BR 4  STRAIN BR 16

PINK LABEL      BLUE LABEL

BROTHERX SISTER                I              I               I

BROTHER X SISTER  BROTHERXSISTER  BROTHERX SISTER

F3

FIG. I.-Scheme of breeding from tumour-bearing and tumour-free hybrids.

1947)), and from I female, BR16, which had a lung tumour but no discernible
mammary tumour when killed at the age of 130 weeks.

Mouse BR6 was mated with a sibling male to provide the F2 generation in

the strain BR6. Mouse BR4 was mated firstly with a brother to start a strain
designated BR4 (pink label), and secondly with an unrelated BR F , male, No. 1,
to start the strain BR4 (blue label). Male No., I also sired the tumour-free
female to start the strain BR 1 6. Fig. I shows the provenance of the four strains.

Each strain was continued by brother-sister matings. The F2 females bred

normally and raised their litters. Most of the P. females raised one litter and
many raised two or three litters; subsequently they were subjected to forced
breeding. Selected F4females raised one or more litters to continue the strains,
but MoSt F4 females were subjected to forced breeding throughout their
reproductive lives. All the mice were similarly housed and fed.

Pedigrees of all mice were kept and the record included a note of the coat
colour ; the BR F hybrids were all of brown or " wild " colour and their progeny
were classified as wild," " white " or " black," minor variations being ignored.
Selective breeding in the F4 generation established " wild," " white " and

OQQ

AO.P%P

AGENT IN MAMMARY TUMOURS IN HYBRID NaCE

black " sublines which with rare exceptions, have bred true to type as far as
the F8 generation.

The results for the first three generations 4hybrid generations F,7F,) are
given in Tables I-IV. The " effective total "' is the number of mice which
surv-ived to the age at which the earfiest tumour was detected in any of the

groups, namely I 10 days in an F4 mouse of strain BR4 (blue label). The

Cc average tumour age " is the average age at which a t-umour was first detected,
the mice being inspected twice weekly. The mice ahve with tumours include
mice with nodules growing and regressmg m repeated cycles; as described by
Foulds (1949b), mice with these nodules almost always die with large typical
tumours. Mice in which nodules were present at some period but not at the

T"LE I.-Strain BR4 (Pink Labd).

Tumour-free.          Tumour-bearin,-.

D"O.       ANve.       Dead.      Alive.                   Average

Hybrid  Effective   A                                  A       Total  Turnour  tumour

incidenec

generation. tow.     Average     Average    Average     Average tumoum         age

No. age at  No.  age   No. age at -NO.   age                 (weeks).

death      (weeks).    death      (weeks).
(weeks).               (weeks).

F2       4   . I . 28   . 0           3 . 62   . 0 -           3      75      62
F3      37   . 15 . 34  . 0          22 . 48   . 0 .          22      59      36
F4      109  . 19 . 30  . 9     69    79 . 47  . 2 . 60       81      74      36

TABLE IL-Strain BR4 (Blue Label).

Tumour-fr".               Tumour-bearhi&

Dead.        Alive.        Dead.        Ahve.

Effective     -A            A            A             A        ToW

EL    tow.       Averw         Average      Averw         Average tumour&

No.   age at  NO.   age    No.   age at  No.    age

death        (weeks).      death        (weeks).
(weeks).                   (weeks).

6   .  2 .  42     .  0           4 .  61     . 0             4
52   . 13 .  33     .  4 .  70    33 .  59     . 2    69      35
101   .  4 -  38     . 19 .  60    69 .  47     . 9    65      78

Tumour Average
incidence tumour

M).         am

(weeks).

Hybrid

geDeratioio

F2
F3
F4

67 51
67 35
77 35

TABLiE: M.-Strain BR6.

Tumow-fr".                Tumow-bearing.

Dead.        AUve.         Dead.        Alive.
Hybrid  Effective

generation. totaL        Average       Average      Average       Average

No.   age at    No.  age         age at  No.    aw

death        (weeks).       death       (weeks).
(weeks).                    (weeks).

F.         4   .  2 .   34     .  0 . -     .  2 .  58    . 0

F;        34   -  8 .   35    .  I .  55    . 23 .  53    . 2    56
F4        98   . 11 .   30     .  6 .  59   . 75 .  50    . 6    58

TUMOUT Average
Total incidence tumour
tumours.   M.        age

(weeks ).

2     .  50      50
25     .  74   -  39
81     .  83   -  35

T-A--iBLF, IV.-Strain BRI6.

Tumow-fr".                 Tumour-besrirW,

Dead.                       Dead.         Artve.

lybrid  Effective       A             A             A             A                 TUMOW

Total  iikeidence
ikeration. total.      Average                      Averan                            (0/).

Average                     Average tumours.     /O
0.  age at   NO.    we      No. age at    Nn.     age

death         (weeks).       death         (weeks).
(weeks).                     (weeks).

F2         10      2 .   54      8 .   89      0 .        . 0   ,           0         0
F3         97   . 14 .   43     - 80 -  66     2 .   68     . I  .  79      3         3
F4       136   . 14 .    26     .120 .  62     I .   54   . I   .  69       2         I

Average
tuinour

age

(weeks).

67
59

H
gem

234

L. FOULDS

time of death are recorded as tumour-free. Many of the mice recorded as dead
without tumour succumbed to epidemic infection about a year after the beginning
of the experiment.

Tables 1-111 show that the incidence of tumours in the strains BR4 (pink
label), BR4 (blue label), and BR6 was unequivocally " high." The three strains
were substantially alike. Tumours developed, indiscriminately as it seemed,
in 11 wild," 11 white," and " black " mice in the F?-F4generations and continue

to develop in " white " and " black " sublines up to the F6 generation. The
later age-incidence of tumours in the F2 females was probably attributable to
the breeding history; the F2 females, in contrast with later generations, were
not subjected to forced breeding.

The incidence of tumours in the BR16 strain (Table IV) so far is extremely
low. The great majority of the mice survive and more tumours may yet develop,
but the average age is already higher than th'e average age of tumour development
in the other three strains. Whatever the final incidence, the contrast between
strain BR 1 6 -and strains BR4 (pink label), BR4 (blue label), and BR6 is
unmistakable.

Mammary tumour agent in tran8planted BRI tumour8.

Extracts of transplaiited tumours of the hormone-dependent BR I strain
(CRI in the previous report by Foulds (1947)) were injected into female BA F,
mice (C57 black   x A      According to previous experience in this laboratory
(Gye, 1948), BA P, hybrids do not develop mammary tumours spontaneously
although other laboratories record a low incidence; mammary tumours develop
when tumour agent is supplied by foster-nursing or by injection.

The BA F, hybrids were 33-46 days old at the beginning of the experiment.
Litter mates were -allotted as equally as possible to three groups ; one group
received extracts of BRI tumours; another group received extracts of
spontaneous mammary tumours froih mice of high-tumour strains; the third
group received no injections. Tumours were ground in -a mortar with sand
and saline to make suspensions which ranged in strength, in different experiments,
from 13 to 20 per cent W/V. The debris was removed by spinning in an ordinary
centrifuge, and the supematant fluid was injected, at weekly intervals, into the
dorsal subcutaneous tissues, each dose being 1-0 c.c. Sixteen mice ree eived
each 4 injections of extracts of BRI tumours of the first or third transplanted
generations, five received 3 injections and two received 2 injections. Twelve
mice received each I injection of an extract of a primary spontaneous C3H tumour
and 3 injections of primary spontaneous R3 tumours, and seven mice received
3 injections of the R3 extracts only. All the extracts were similarly prepared.
The females were caged with males and subjected to forced breeding, litters
being removed within 48 hours (usually within 24 hours) of birth.

Table V summarizes the results at 89 weeks from the beginning of the experi-
ment. The " effective totals " comprise the mice which survived to the age
when the first tumour was detected (180 days in a mouse injected with BRI
extract). Tumours developed with comparable frequency in the two groups
which received tumour extracts and at similar times; none developed, within the
period of observation, in the control mice, I I of which survived, without apparent
tumours at ages of 92-96 weeks. The extracts were iiot certainly free from living

" R
AGEN"r IN MAMMARY TU OUM          IN EEYBRM  MICE         lfiftfto

cefls, but the pomibility that the t aoun resulted from cell transplantation
was negligible, being dismunted by the development of tumoun in mamnitary
timue at sites remote from the point of injection, by the long intervals between
the injectioxw and the appearance of tumours, and by the genetic difference8
between the mice providing the extracta and the mice which developed tumours.

TABix 'V.-Tunwur Incidewe in Injezkd and Normal BA F I Mice.

Dead           Ahw            D"kd
wiuxmt         wtuwut          Wm
tunmur.       tunww.          tumow.

Experimmt.        Effecu"                       A     It r           -1

to"L           Average        Average        Average

NO.     am      NO.     age    NO. tamour age

(weeks).       (weeks).       (weeks).
BRI tumour extract       21      2      55       0              19     48

(90-5%)

Spontaneous tumour extract  17   3      66       0              14     43

(824%)
NOMW controls            12      I      82      11      94      0

The rmults inclicated that transplanted BRI tumours contained mammarv
tumour agent comparable in activity with that in spontaneous growtim in
mammary tumour strains.

Spontaneom mammary tumour8 in RB F , mice.

A colony of RB F, mice (R3 ? x C57 black d) was maintained to compare
the tuinour incidenm with that in BR F, hybrids. The mice were subjected to
forced breeding like the BR mice and similarly housed and fed. Of the effective
total of 36 females, 33 (91-7 per cent) developed amma-ry tumours. The average
number of tumours per mouse was 2-5, 24 mice having from 2 to 6 t aours each.
The average age at the first detection of a t aour was 25 weeks; the earhest
tumour wa-s recorded at the age of 17 weeks, and the latest at 54 weeks.

The 3 mice without tumours are alive at ages of 81, 81 and 85 weeks respec-
tively. It is noteworthy that although continuously with males they have had
no litters for about a year ; previoxwly litters were not recorded. No observations
were made on virgin mice.

Incidence of tumours in (C57 black ? x ageW-free R3 CT) F]L hybri&.

Remale mice taken at random from the laboratorystock of C57 black were
mated with males:firom a colony of R3 mice free from milk agent. I am indebted
for the male mice to my coReague Dr. B. D. PuWmger, who raiwd the colony
from a fitter of R3 mice removed from their mothers unediately after bftth
and suckled by C57 black foster-mothers. Up to the prewnt only 2 a      ical

mary tumoun, of squamous type, have developed at the age of 23 months
amonpt 117 breeding females over 9 months old (Pullinger, 1947, and
penonal communication). The F,, hybrids were subjected to forced breeding
throughout their reproductive lives.

One hundred female hybrids were reared; 21 died without tumours at an
average age of 21 weeks and 79 are alive at an average age of 57 weeks. Of the

mice, 29 are lem thain a year old (average 39 weeks) and 50 are over a
year old (average 67 weeks). The only t riour encountered so far ww detected
at the age of 64 weeks in a mouse which survives ; the str cture of the tumour

236

L. FOULDS

is unknown. Up to the present therefore the incidence in mice over a year old
is 2 per cent.

Of the 23 tumours found in BR P, hybrids with normalR3 fathers, 20 were
first seen in rnice less than a year old, the average age at detection being 38 weeks'
The incidence in BR F, hybrids with agent-free R3 fathers is much lower or
much later.

Table VI summarizes for easy comparison the incidences of mammary
tumours in the various hybrids.

TABLIF, VI.-Summary of Tumour Incidences.

Effective   Tumour     Average

total.    incidence  tumour age

(%).       (weeks).
BR F,                                           71         15         40
BR F,, (Agent-free cl)                          50          2         64
RB F,                                           36         92         25
Strain BR4 (pink label)  F                      14         64         55

VI, BR4 (blue V.      F3                    123          67         39

BR6               F4                     308         77          35

F2                      10          0

Strain BR16             F3                      97          3         67

F4                     136          1         59

DISCUSSION.

Andervont (1945a) reviewed the records of relatively high incidence of
mammary tumours in hybrid mice having low-tumour strain mothers, and hence
presumed deficient in the mammary tumour agent. The possible explanations
of the. phenomenon were speculative because tests for the presence of tumour
agent in the hybrids were not 'made. Subsequently, Andervont (1945b) and
Andervont and Dunn (1948, a, b, c) investigated the occurrence of tumours in
mice with strain C (low-tumour) mothers and strain C3H (high-tumour) fathers.
Their results, which corresponded in some respects with my observations on
hybrids with C57 black (low-tumour) mothers and R3 (high-tumour) fathers
but differed in other respects, are discussed in later paragraphs.

The incidence of tumours in BR F, hybrids (C57 black ? x R3 S) resembled
that recorded by others for various hybrids with low-tumour strain mothers
and high-tumour strain fathers. Although the incidence was not high the
tumours appeared at an average age of 38 weeks, which was within the range of
age incidences of tumours in high-tumour strains. The histological structure
was unremarkable except in two tumours which developed near the sites of
injection of a carcinogen and in one tumour in a virgin. The sex-limited
transplantation described in a previous paper was not pecuhar to BR F 1 hybrids ;
as recorded in the paper which follows (Foulds, 1949a), some tumours in the
reciprocal RB F 1 hybrids, which had a high incidence of tumours, behaved
similarly. Mammary tumour agent, comparable in activity with the agent
present in spontaneous tumours of high-tumour strain mice, was demonstrated
in a transplanted BR F., tumour. The BR F tumours, therefore, differed
con-sistently in no particular from the typical mammary tumours of mice of
inbred high-tumour strains. The tumours, however, were concentrated in a
minority of litters of BR F, niice, the majority of families having none so that

the total incidence was relatively low. Observations on the F2 and subsequent

237

AGENT IN MAMMARY TUMOURS IN HYBRIID 3HCE

generations confirmed the imphcation that the BR F , hybrids were hetero-
geneous.

The descendants of the two tumour-bearing BR F 1 females had a

incidence of tumours at a relatively early age, and resembled in all respects mice
of inbred     -tumour strains.  The       incidence was maintained at least

to the F,, or F6 generations. The diversity of genetic constitution in the F2

and subsequent generations did not noticeably affect the incidence of tumours
which developed indiscriminately in " black," " white " and " wild " mice
and in each of the " black, ly 31 4 'white" and " w-fld " subhnes estabhshed by
selective breeding. By contrast the tumour incidence in the progeny of a
tumour-fi-ee BR F 1 female sired by a male that sired, also, one of the

tumour strains was extremely low or extremely late. These results recaRed
Bittner's (I 940) observations on strain A mice nursed by C57 black foster-mothers.
A minority (7-9 per cent) of the fostered A females developed mammary tumours,
presumably because they received tumour ageDt from their strain A mothers
before being transferred to foster-mothers, and the tumour incidence in their
progeny was high (58-5 per cent). The majority of the fostered A mice developed
no tumours and the incidence in their progeny was low (6-1 per cent).

In sum, the observations indicated that a minority of (C57 black  x R3

F]L hybrid females carried and transmitted the mammary tumour agent and
developed mammary tumours, whereas the majority of the mice did not carry
or transmit the agent and did not develop tumours. Direct and indirect evidence
for the presence of tumour agent in tumour-bearing hybrids limits discussion
to the source of the agent and the reason for its uneven distribution amongst
the F , hybrids.

The subhnes of C57 black mice maintained in various laboratories are not
now identical and some possibly carry the mammary tumour agent, perhaps
in attenuated or masked form. In this laboratory mammary tumours have
not been recorded in the pedigreed stock although they have occurred, rarely,
in unpedigreed mice derived from the C57 black stock as, for example, by random
interbreeding; our subhne maintains therefore the essential characteristics of
the original stock. Mamm ry tumours have developed in two types of hybrid
with C57 black mothers, namely (C57 black x R3) F, and (C57 black x C3H)
F, but not in a third type (C57 black x A) F, (Gye, 1948, and this paper).
Evidently the strain of the father had a subsUntia'l effect on the incidence of
tumours in F IL hybri&-,. The patemal contribution was not probably genetic
for, as shown in this paper and in previous reports from this laboratory (Dmo-
chowski, 1944, 1945), the C57 black x A hybrids developed tumours abundantly
when tumour agent was supphed to them artificiafly. The uncompleted
observ-ations on hybrids having fathers belonging to a subline of R3 mice deprived
of tumour agent accorded with similar observations bv Anderv-ont and Dinnn
(1948a, b, c) in showing a substantial reduction in the incidence of tumours

comparison with similar hybrids whose fathers derived from a line carrying the
tumour agent. The experiment was open to the criticism, which Anderv-ont
and Dunn apphed to their ow-n experiment, that the agent-fiee and agent-
bearing malet; were separated by several generations of inbreeding and possibly
differed geneticaRy. With this reserv-ation, both experiments indicated a
transmission of tumour agent by males of high-tumour strains to their hybrid
offipring. The probabihty of this interpretation was increased by Anderv-ont

238

.L. FOULDS

and Dunn's (1948a) demonstration of tumour agent in the seminal vesicles
of male mice of high mammary tumour strains. Genetically, the BR F 1 hybrids
were uniformly susceptible to the development of mammary tumours, as shown
by the high incidence which resulted from artificial administration of tumour
agent (Dmochowski, 1946), and by the high natural incidence now recorded in
the reciprocal RB F, hybrids. The relatively low natural incidence of tumours
in BR F 1 was attributed provisionally, therefore, to -the erratic transmission of
tumour agent by male parents to their hybrid offspring.

Andervont (1945b) and Andervont and.Dunn (1948a, b, c) studied the hybrid
offspring of females of the strain C, having a mammary tumour incidence of less
than I per cent, and males of the high-tumour strain C3H. Tumour agent was
.not demonstrable in the tumours which developed in the hybrids nor in lactating
mammary glands of strain C females. No evidence was found, in any experiment,
of transniission of tumour agent by strain. C females. Tumour agent was
demonstrated, however, in the.seminal 'vesicles of male rnice of high-tumour
strains, and the incidence of tumours in (C  x C3H S) F, hybrids was greatly
reduced when the male present derived from a line of C3H nlice lacking the
tumour agent.

The C x C3H hybrids apparently differed from the BR F, hybrids in several
important particulars: the incidence of tumours was much -higher in C x C3H
hybrids (about 60 per cent) than in BR F 1 hybrids (about 15 per cent), yet the
average age at the appearance of tumours was much later (about 22 months
compared with about 10 months); the C x C3H tumours were not concentrated
in particular farnilies as were the BR tumours, and tumour agent was not
demonstrable in the tumours. Andervont and Dunn's most recent publication
(1-948c), received after the present investigation was completed, showed, however,
that a minority of tumours in C x C3H hybrids corresponded with the tumours
now described in BR F, hybrids. In general the (C x C3H) F, hybrids had the
characteristics already enumerated ; they, had a high incidence of tumours at
a late average age and not concentrated in particular families. Tumour agent
was not demonstrable in the hybrids or in their tumours, and the offspring of
tumour-bearing mice, whether obtained by back-crossing or by brother-sister
matings, rarely developed tumours. Two litters of -(C x C3H) F, were excep-
tional in that tumours developed in sisters at unusually early ages (4-10 months).
The backcross offspring of two tumour-bearing females of one litter all developed
tumours at average ages of 7 and 9 months respectively. The 4 tumour-bearing
females of the other litter were mated with a brother, and breeding was continued
by brother-sister matings. A tumour incidence of 85-100 per cent at ages of

5-10 months was observed in each generation as far as F6. The presence of

tumour agent was demonstrated by using hybrids from each of these lines as
,foster-mothers for susceptible mice. Tests for agent in the tumours were not
reported. Evidently the exceptional " early " tumours in (C x CM) F, hybrids
were the counterparts of the tumours now described in BR F., hybrids.

It ik concluded that a minority of BR F, hybrids carry a tumour agent,
derived probably from the male parent, and develop tumours at a relatively
early age, whereas the majority of the hybrids receive no tumour ag .ent and
remain free from tumours. The heterogeneity of the F, hybrids is attributed,
provisionally, to erratic transmission of tumour agent by the male parent.
Apparently a smaller minority of the hybrids used by Andervont and Dunn

AGENT IN MA MMARY TUMOURS IN HYBRID NaCE                  239

develop early tumours exactly comparable with the BR F , tumours, but tumours
develop in a high proportion of the remaining mice about a year later. The
late tumours in which tumour agent has not been demonstrated account for the
exceptionaHy      tumour-incidence in these particular hybrids. The present
investigations provide no clue to the nature of the late tumours. It is evident.
however, that the (C-X OR) F, hybrids, like the BR F, hybrids, are hetero-
geneous.

It is noteworthy that experimental deprivation of tumour agent greatlv
reduces but doe6 not completely suppress the development of mamm ry tumours.
My observations add two examples, namely the fine BR 1 6 established by breeding
from a tumour-fi-ee hybrid female, and the colony of F, hybrids derived from
C57 black females and R3 males deprived of milk agent. The aetiology of these
r. .1 residual " tumours, which possibly corresWnd with the late tumour-s in
(C x C3H) FjL hybrids, is obscure.

SUMMARY.

Typical mamm ry tumours developed in about 15 per cent of the breeding
females of the first generation hybrids, Ca-7 black Y x R3 3 at an average age
of 40 weeks. The incidence of tumours in the reciprocal hybrids, R3 Y x CO
black 3, was 92 per cent at an average age of 25 weeks.

Mammary tumour agent was demonstrated in a transplantable tumour which
originated in a (C57 black 9 x R3 (3) F, mouse by injecting tumour extracts
into (C57 black Y x A 3) F ]Lhybrids which, otherwise, did not develop mamm ry
tuirnours. The progeny of two tumour-bearing (C57 black Y x R3 cT) -P., females
had a high incidence of mammary tuinours (about 70 per cent)            ed
through at least 5 generations. By contrast the progeny of a tumour-fi-ee female
had a low incidence (0-3 per cent) similarl         . It was concluded that-
the F, kvbrids were heterogeneous; a mj'nority, concentrated in certain littem,
developed mamm ry tumours, carried the mammary tuinour agent and trans-
initted the agent to their offspring, which had a  incidence of tumours; the
majo     did not develop tumours and did not transmit the tumour agent to
their off-spring, which, with rare exceptiom, did not develop tumours. Since
the incidence in (C57 black Y x R3 3) F, hybrids was reduced or delayed when
the male parent derived from a line of R3 fi-eed from tumour agent, the low or
moderate incidence of mammary tumours in hybrid mice with low-tumour strain
mothers and      -tumour strain fathers was attributed, provisionaRy, to the
erratic transmission of tumour agent by the male parents.

REFERENCES.

A.-,mimvoN_T, H. B.--(1945a) " Symposium on Mammary Tumours in Mice," Amer. Am.

Adv. Sci., 22, 123.--(1945b) J. nat. Cancer Inst., 5, 391.

ldemANDDrNN, T. B.--(1948a) Ibid., 8, 227.--(1948b) Ibid., 8, 235.--(1948c) Ibid., 9,

89.

Brrr--%-ER., J. J.--(1940) Ibid., 1, 155.

DmociEiowsKi, L.--(1944) Brit. J. e-xp. Path., 25,138.-(1945) Ibid., 26, 192.--(1946)

Ibid, 27, 391.

FouLDs. L.--(1947) Brit. J. Cancer, 1, 362.-(1949a) Ibid., 3, 240.--(1949b) Ibid.,

in press.

GYE, W. E.---(1948) Ann. Rep. Imp. Cancer Reg. Fd., 45, 3.
PUIJJNGJM, B. D.--(1947) Brit. J. Cancer, 1, 177.